# Outcomes of Patients with Non-Small Cell Lung Cancer and Brain Metastases Treated with the Upfront Single Agent Pembrolizumab: A Retrospective and Multicentric Study of the ESCKEYP GFPC Cohort

**DOI:** 10.3390/curroncol31030126

**Published:** 2024-03-21

**Authors:** Simon Nannini, Florian Guisier, Hubert Curcio, Charles Ricordel, Pierre Demontrond, Safa Abdallahoui, Seyyid Baloglu, Laurent Greillier, Christos Chouaid, Roland Schott

**Affiliations:** 1 Department of Oncology, Institut de Cancérologie Strasbourg Europe, 67200 Strasbourg, France; s.nannini@icans.eu (S.N.); s.abdallahoui@icans.eu (S.A.); 2 Department of Pneumology, UNIROUEN, LITIS Lab QuantIF Team EA4108, CHU Rouen, Normandie University, Rouen and Inserm CIC-CRB 1404, 76000 Rouen, France; 3 Department of Pneumology, Centre Régionale de Lutte Contre le Cancer François Baclesse, 14000 Caen, France; 4 Department of Pneumology, Centre Hospitalier Universitaire, 35000 Rennes, France; 5 Department of Radiological, Centre Hospitalier Universitaire de Strasbourg, 67200 Strasbourg, France; 6 Multidisciplinary Oncology and Therapeutic Innovations, APHM, INSERM, CNRS, CRCM, Hôpital Nord, Aix Marseille University, 13015 Marseille, France; 7 Department of Pneumology, Centre Hospitalier, 94000 Créteil, France

**Keywords:** non-small cell lung cancer, brain metastasis, immunotherapy, real-life data, central nervous system

## Abstract

Non-small cell lung cancer (NSCLC) is the most common cause of brain metastasis (BM). Little is known about immune checkpoint inhibitor activity in the central nervous system, especially in patients receiving monotherapy for tumors with a tumor proportion score (TPS) ≥ 50%. This noninterventional, retrospective, multicenter study, conducted with the GFPC, included treatment-naïve patients strongly positive for PD-L1 (TPS ≥ 50%) with BM receiving first-line single-agent pembrolizumab treatment between May 2017 and November 2019. The primary endpoints were centrally reviewed intracranial overall response rates (ORRs), centrally reviewed intracranial progression-free survival (cPFS), extracranial PFS, and overall survival were secondary endpoints. Forty-three patients from five centers were included. Surgical or local radiation therapy was administered to 31 (72%) patients, mostly before initiating ICI therapy (25/31). Among 38/43 (88.4%) evaluable patients, the intracranial ORR was 73%. The median PFS was 8.3 months. The cerebral and extracerebral median PFS times were 9.2 and 5.3 months, respectively. The median OS was 25.5 months. According to multivariate analysis, BM surgery before ICI therapy was the only factor significantly associated with both improved PFS (HR = 0.44) and OS (HR = 0.45). This study revealed the feasibility and outcome of front-line pembrolizumab treatment in this population with BM.

## 1. Introduction

Lung cancer, including non-small cell lung cancer (NSCLC), is the most common cause of brain metastasis (BM), as 20 to 40% of patients with NSCLC will develop BM during the clinical course of their disease [1]. Brain involvement at initial staging is estimated to occur in approximately 10% of patients with NSCLC according to large retrospective cohorts [2]. Moreover, NSCLC is historically associated with poor life expectancy, especially in the case of BM, leading to frequent exclusion of these patients from clinical trials [3,4]. However, in recent decades, substantial improvements have been made in modified NSCLC management, although limited data are available for patients with BM. In advanced NSCLC without targetable molecular alterations, validated first-line treatment is now based on monotherapy or combination therapy with immune checkpoint inhibitors (ICIs) that target programmed death 1 receptor (PD-1), its ligand (PD-L1), or cytotoxic T-lymphocyte-associated protein 4 (CTLA-4) [5,6,7]. Little is known about the efficacy of ICIs against BM, and the central nervous system (CNS) is still considered a sanctuary of immune privilege because of limited penetration of systemic therapy through the blood–brain barrier [8,9]. Treatment recommendations for BM include local therapies such as surgery, stereotactic radiation therapy (SRT), or whole-brain radiotherapy (WBRT), despite several induced toxicities [10,11,12]. In a pooled analysis of the KEYNOTE-021, -189, and -407 trials, which validated the overall survival (OS) benefit from the combination of pembrolizumab and platinum-doublet therapy, the improvement was reversed in patients with BM, with a hazard ratio of 0.48 and an absolute benefit of 11.2 months. However, only patients with stable and asymptomatic BMs were included [5,6,13,14]. To our knowledge, only one prospective study has explored the efficacy of monotherapy ICIs in patients with NSCLC and untreated BMs [15]. Among thirty-four patients with an expression of PD-L1 of at least 1%, nearly 30% had a BM response, including seven patients with partial response (PR) and four with complete response (CR). No difference was detected when PD-L1 expression was lower than 1%. According to a recent multivariate analysis of patients with untreated, active, or unstable BMs, brain involvement was not associated with poorer outcomes in patients treated with ICIs [16]. The GFPC-ESCKEYP cohort is a large national multicentric cohort that aims to report real-word data for advanced NSCLC patients with high-level PD-L1 positivity, defined as those with a tumor proportion score (TPS) ≥ 50%, for whom first-line treatment with pembrolizumab as a single agent was initiated. The results revealed progression-free survival (PFS) and OS rates similar to those in pivotal studies [17]. On the basis of the findings of this real-world study, we report here the data for patients with BM at diagnosis to assess the efficacy of first-line pembrolizumab in this specific population.

## 2. Materials and Methods

### 2.1. Study Design and Patients

We extracted data for patients with BM at the start of pembrolizumab, treated at five different centers and enrolled in the ESCKEYP study from 5 May 2017 to 22 November 2019. Patients must have been treated by pembrolizumab monotherapy, without chemotherapy, in first line for advanced NSCLC with high-level PD-L1 positivity, defined as a TPS ≥ 50%. Patients could have been treated earlier for localized NSCLC. Data on sociodemographic (age, sex, comorbidities), clinical (performance status, smoking status), pathological (histology, PD-L1 expression), molecular (mutational status), BM (localization, timing to diagnosis, imaging assessment, size, number, radiological characteristics), extracranial (number of metastases, sites), and associated treatment (surgery, radiation therapy) characteristics were retrospectively collected from medical records. BM at inclusion was assessed using computed tomography (CT) or magnetic resonance imaging (MRI), and might be asymptomatic, pretreated or not. Pembrolizumab was administered intravenously at a dose of 200 mg every 3 weeks until tumor progression, non-manageable toxicity, or death.

### 2.2. Assessments

Radiological assessments of brain and extracranial disease were systematically centrally reviewed using Response Evaluation Criteria in Solid Tumors (RECIST) 1.1 for brain and extracranial response as well as the RANO-BM criteria for BM. The percentage of necrosis was calculated as the ratio of the necrotic area to the tumor area. The study was conducted in accordance with the Declaration of Helsinki and Good Clinical Practice Guidelines, and was approved by a national independent Ethics Committee (2019-A02073-54; 11 December 2019).

### 2.3. Outcomes

The primary endpoint was the intracranial and extracranial overall response rate (ORR), which was defined as the percentage of patients who achieved intracranial and extracranial partial (PR) or complete (CR) response according to Response Evaluation Criteria in Solid Tumors (RECIST) 1.1. The intracranial ORR was also assessed according to the RANO-BM criteria. As secondary endpoints, we assessed the following: centrally reviewed intracranial progression-free survival (cPFS), defined as the time from the start of pembrolizumab treatment to disease intracranial progression according to the Response Evaluation Board (RECIST) 1.1 or death from any cause; PFS, defined as the time from the date of pembrolizumab start to the date of first disease progression or any-cause death; OS, defined as the time from the start of follow-up to the date of death from any cause and determined at the date of last contact or cutoff date (18 January 2021); and the safety and toxicity of pembrolizumab according to the Common Terminology Criteria for Adverse Events (CTCAE) version 4.0.

### 2.4. Statistical Analysis

Patient characteristics are described using numbers and proportions for categorical variables and means, SDs, medians, and IQRs for continuous variables. PFS and OS were determined using the Kaplan–Meier method. Univariate Cox models were applied to select the most promising prognostic variables for PFS and cPFS, which included PD-L1 expression, radiotherapy (yes or no), surgery (yes or no), number of metastases, the ratio of the sum of the size of BM to the number of BMs, necrosis (more or less than 50%), smoking status (no smoker versus ancient or former smoker), PS, body mass index, and reported side effects related to the ICI (yes or no). A multivariate Cox model was then applied to adjust for potential confounders. Multivariate analysis was conducted by including relevant clinical variables, with cPFS and then PFS as the dependent variable and prognostic factors as the explanatory variables. Potential confounding criteria of interest included in the multivariate analysis were surgery, number of metastases, the ratio of size over number of BM, smoking status, PD-L1 expression, age, and PS. Hazard ratios (HRs) with their respective 95% CIs and *p* values were calculated. A *p*-value < 0.05 was considered to indicate statistical significance. Statistical analyses were performed using R statistical software (version 4.1.2; R Foundation for Statistical Computing).

## 3. Results

### 3.1. Patients and Tumor Characteristics

Forty-three patients with BM were included from five centers. Five of them were already treated for local NSCLC. The median age was 64 years (44 to 84) years. Twelve patients (28%) had a performance status ≥ 2, five (11.6%) were never smokers, and thirty-four (79%) had adenocarcinoma. Fourteen (32%) patients had *KRAS* mutations. Thirty-seven (86%) patients had extracranial metastasis. None of the patients had proven meningeal involvement. PD-L1 expression was greater than 75% in 22 (51%) patients (Table 1).

### 3.2. Characteristics of Brain Metastases

The diagnosis of BM was based on MRI in 23 (53.5%) patients and CT in 20 (46.5%) patients (Table 2). For five (12%) patients, BM was diagnosed as metachronous to an initially localized NSCLC. Twenty-eight patients (65%) had symptomatic BMs (headache, epilepsy or neurological defects). After central review, the median number of BMs per patient was two (1–12). Localization was well balanced between each brain area (parietal, temporal, frontal, occipital, or infra-tentorial). The median size of the largest BM was 19.3 (4.4–41.1) mm, with a median area of edema of 14.2 (0.3–87.9) cm^2^. The extent of necrosis on the largest BM was < 25% for 16 (41%) patients and greater than 75% for 14 (36%) patients (Table 2).

### 3.3. Local Management of Brain Metastasis

Local therapy, either surgical or local radiation therapy, was administered to 27 (63%) patients (Table 3); 18 (42%) patients received surgery, including 8 (19%) with incomplete surgery. Radiotherapy was delivered for 25 (58%) patients: 19 (44%) received radiotherapy in stereotactic condition (SRT), including 11 (26%) in a postoperative setting. Whole-brain radiotherapy (WBRT) was performed for 5 (12%) patients. Twelve patients (28%) underwent sequential cerebral radiotherapy followed by ICI therapy, with a median delay before the start of ICI therapy of one week. Twenty-five (58%) patients were locally treated before initiation of ICI therapy. Six (14%) patients experienced progression or died before new imaging but after start of ICI therapy. Two (4%) patients underwent radiotherapy following the first cerebral progression during ICI therapy and continued pembrolizumab treatment for more than 3 months before extracranial progression.

### 3.4. Outcomes

The median time between advanced NSCLC diagnosis and the start of ICI therapy was 36 (3–132) days, with a median number of cycles before the best response to ICI therapy of 5 (1–13). cORR was 65% (CR and PR in 15 (34.9%) and 13 (30.2%) patients, respectively). An additional 3 (7.0%) patients had stable disease, leading to a cerebral disease control rate of 72% in the whole cohort; 12 (27.9%) patients had progressive disease as the best response (Table 4). As mentioned, 16 (37.2%) patients did not have local therapy and were treated with pembrolizumab alone. In this part of the cohort, six (37.5%) patients had a cerebral CR, four (25%) had a PR, and one (6.2%) had an SD. Five (31.3%) patients had a progressive disease or died before a new cerebral imaging. 

**Figure 1:**
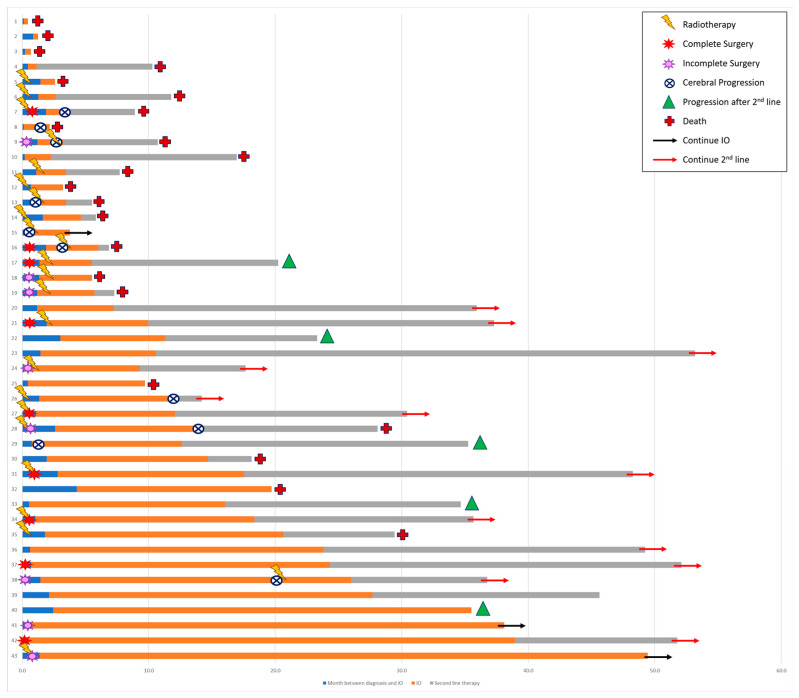
Brain medical management.

For patients from the whole cohort with nonprogressive disease, the median shrinkage of the BM after the start of ICI therapy was 51%, while that of the brain edema was 82% (Table 4). The patterns of brain progression were new BM in two (20%) patients, progression of existing BM in four (40%) patients, and both in four (40%) patients (Figure 1).

**Figure 2:**
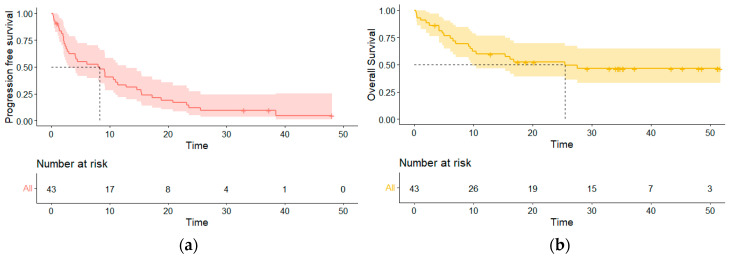
Survival curves of the entire population. (**a**) Progression-free survival curve; (**b**) overall survival curve.

The median PFS of the cohort was 8.3 (95% CI 3.0–13.8) months. Among the 26 patients with progression, 23 had a dissociated response with 16 (61.5%) that had systemic progression only, and 7 (26.9%) had cerebral progression only. Patients with cerebral progression first had a median cerebral PFS of 5.3 (95% CI 3.0–14.7) months. Patient with systemic progression first with or without cerebral PD had a median systemic PFS of 9.2 (95% CI 4.5–17.3) months. The median OS was 25.5 (95% CI 9.8-NR) months (Figure 2).

Twenty-four patients (56%) reported an IrAE, but none were higher than grade 3 according to the CTCAE. IrAEs were reported after a median of 5 cycles, and the most frequent IrAEs were asthenia (42%), diarrhea (38%) and thyroiditis (25%). The main reasons for pembrolizumab interruption were progression or death in 18 (67%) patients and adverse events in 4 (15%).

### 3.5. Following Therapy

After definitive discontinuation of pembrolizumab, 22 (62%) patients underwent additional treatment. Eleven (31%) patients received local therapy, such as BM or extracerebral radiotherapy, and seven of them also started new systemic treatment. Sixteen patients (42%) received chemotherapy as a new systemic line, and two patients started targeted therapy. On this second line of treatment, no patient achieved systemic CR, but seven (39%) achieved PR, and the second median PFS was 10.8 months (Table S1).

### 3.6. Prognostic Factors for Cerebral Progression

According to univariate analysis of potential prognostic factor for cPFS, only the ratio of cumulative size over the number of the BM was associated with cPFS (HR = 1.08, *p* = 0.006). Surgery, radiotherapy, or the number of metastases were not correlated with cPFS (Table S2). The ratio value was also the only variable significatively associated with cPFS in the multivariate analysis (HR = 1.11, *p* = 0.006).

### 3.7. Prognostic Factors for Systemic Progression

According to univariate analysis, surgery of the BM was significantly associated with improved PFS (HR = 0.44, *p* = 0.015). IrAEs (HR = 0.53, *p* = 0.051) and performance status (HR = 1.30, *p* = 0.09) showed some tendency, but analyses were not significant (Table S3). According to multivariate analysis for PFS, surgery for BM was associated with improved PFS (HR = 0.33, *p* = 0.011), and so is the ratio of cumulative size over number of the BM (HR = 1.05, *p* = 0.018) (Table 5).

## 4. Discussion

In this retrospective and multicentric study based on real-world data, the single agent pembrolizumab was shown to be beneficial as an upfront treatment in patients with advanced NSCLC with PD-L1-positive expression ≥ 50% and BM. The cORR, median PFS, and OS were 65%, 8.3, and 25.5 months, respectively. The median range of shrinkage of brain oedema was 82% [6; 100]. For patients for whom data were available to assess progression, 10/26 (38%) had cerebral progression and 16 (62%) had exclusively systemic progression. 

Most of the patients received local therapy before initiating ICI therapy. Surgery, but not radiotherapy, was associated with a slightly significant improvement in the cPFS. Although this study cannot clearly determine the benefit of ICI therapy without local therapy, we reported prolonged PFS in 16 patients who did not receive complete local therapy, with a median PFS of 8.3 months, similar to that of the overall population. Moreover, six patients had a complete response of their BM only with pembrolizumab. These results support the benefit of pembrolizumab even in the CNS.

These real-world data are consistent with those from prospective studies. In a histology-agnostic phase 2 trial of patients with BM, including seven with NSCLC (of whom two had EGFR mutations and one had an ALK rearrangement), the overall intracranial benefit rate of pembrolizumab was 42.1%, and 43% of patients with NSCLC had an intracranial response, which is consistent with our results [18]. Moreover, the ATEZO-Brain trial reported similar systemic and cerebral efficacy of the addition of immunotherapy to chemotherapy. These data led to the same conclusion about the CNS activity of ICIs [19].

The brain is considered an immune-privileged site where therapeutic drugs cannot penetrate the blood–brain barrier (BBB). This paradigm may change, as recent data suggest that components and interactions of the BBB are altered while BMs develop, compromising BBB integrity and allowing penetrability of ICIs [20,21]. In recent translational studies performing RNA sequencing, advanced metastatic lung cancer seems to present distinct molecular and cellular features from those of the early stage, with sustained reprogramming of the tumor microenvironment (TME) [22]. In the case of BM, the intracranial TME seems to be more immunosuppressive than the extracranial TME is, with a growing interest in immune-based strategies that promote antitumoral T-cell activity [18,22,23,24,25].

Considering the scarcity of data for patients with BM treated with ICIs, this study, based on real-world data, reports descriptive analyses of numerous patients with BM. One strength of this study lies in its real-world setting; most of our patients experienced symptoms (65.1%), and 27.9% had a PS > 2. Interestingly, performance status was not associated with poorer survival outcomes. These findings may support the inclusion of patients with poorer performance status in further studies assessing the efficacy of ICIs.

To our knowledge, only one prospective phase 2 study has been conducted to assess the activity of the single agent pembrolizumab in NSCLC patients with BM. This study included patients with PD-L1 expression ≥ 1% in tumors and non-symptomatic or locally treated BMs [15,21]. The authors reported that 29.7% of PD-L1-positive NSCLC patients achieved a cORR with a 2-year OS of 34%. The 2-year OS was slightly greater in our analysis, in which 41.8% (18/43) of the patients were alive. In contrast to our study, patients with BMs exceeding 20 mm, neurologic symptoms, who required corticosteroids or who had a PS > 2 were excluded, limiting the extrapolation of these results. However, these results are consistent with retrospective observational studies with smaller cohorts of patients [26]. In 2020, a small cohort of 13 patients with BM treated without local therapy but only pembrolizumab-based therapy showed a cORR of 36% [27]. In another setting, the meta-analysis META-L-BRAIN reports data from ICI monotherapy retrospective trials in second line treatment. It reports a cORR of 16.4%, but data about potential complementary radiotherapy or surgery were unclear [28]. Also, compared to our study, these studies were not limited to high-level PD-L1 NSCLC, and could include chemotherapy plus pembrolizumab combination as potential treatment setting. Our trial is the first to describe this treatment in the specific setting of first line monotherapy by pembrolizumab for high-level PD-L1 NSCLC with BM. Nevertheless, prospective data to provide additional evidence for the efficacy of ICIs for the treatment of BM are needed [16].

Our study has several limitations inherent to its retrospective nature and relatively small sample size. All patients (*n* = 43) had a baseline imaging for BM diagnosis, either MRI or CT scan. As in other real-world studies, the timing of radiological examinations was not standardized, and may represent bias due to delays before response evaluation. However, we performed a central review of all radiological examinations to reduce reporting bias, and performed assessments using both the RECIST 1.1 and RANO-BM criteria [29,30]. Despites the small sample size, we successfully showed the potential impact of the surgery of the BM on the PFS. The ratio of the cumulative size of BM over the number of BM seemed to have a real impact on the PFS, but also on the cPFS. Prospective trials on a bigger sample may confirm the impact of these two variables on the outcomes of this population. 

Finally, we did not report any grade 3 or more irAEs. Meta-analysis confirmed that ICI monotherapy is overall better-tolerated than chemotherapy alone. Due to its efficacy and favorable safety outcomes, ICI monotherapy seems to be a reasonable option for treating BM in NSCLC patients [31].

## 5. Conclusions

This study, based on real-world data, supports the feasibility and favorable outcome of pembrolizumab monotherapy as a front-line treatment for high-level PD-L1 (TPS ≥ 50%) NSCLC diagnosed with BM, especially in association with local therapy.

## Figures and Tables

**Table 1:** Patient characteristics (*n* = 43).

Characteristics	Population, *n* (%)
Age (years), Median (range)	64 (44–84)
Sex	
Male	30 (69.7)
ECOG PS	
0–1	31 (72.1)
≥2	12 (27.9)
Smoking status	
Never smoker	5 (11.6)
Former smoker (>1 year)	18 (41.9)
Current smoker	19 (44.2)
Unknown	1 (2.3)
Histology	
Adenocarcinoma	34 (79.1)
Squamous	4 (9.3)
Others	5 (11.6)
Tumor PD-L1 expression †	
50–75%	19 (46.3)
≥75%	22 (51.2)
Mutational status	
KRAS	14 (32.6)
P53	5 (11.6)
BRAF	1 (2.3)
MET	1 (2.3)
ROS1	1 (2.3)
Others	2 (4.6)

Abbreviations: ECOG PS, Eastern Cooperative Oncology Group Performance Status; PD-L1, Programmed Death Ligand 1. Notes: † Status was unknown for two patients.

**Table 2:** Characteristics of brain metastasis (BM) patients (*n* = 43).

Characteristics	Patients, *n* (%)
Imaging for BM diagnosis	
MRI	23 (53.5)
CT-scan	20 (46.5)
Timing of diagnosis	
Synchronous	38 (88.4)
Metachronous	5 (11.6)
Symptomatic BM	
Yes	28 (65.1)
Localization	
Parietal	19 (44.2)
Temporal	15 (34.9)
Frontal	14 (32.6)
Occipital	14 (32.6)
Infratentorial	12 (27.9)
Number of BM, Median (range)	2 (1–12)
Size’s sum of all BM, mm, Median (range)	28.4 (5.0–136.2)
Size of the largest BM, mm, Median (range)	19.3 (4.4–41.1)
Surface of the largest BM, cm^2^, Median (range)	3.1 (0.1–27.2)
Biggest area of edema, cm^2^, Median (range)	14.2 (0.3–87.9)
Necrosis, (*n* = 39) ^†^	
≤25%	16 (41)
25–50%	5 (12.8)
50–75%	4 (10.3)
≥75%	14 (35.9)

Abbreviations: BM, brain metastasis; MRI, magnetic resonance imaging. Notes: ^†^ Four patients did not have data about metastasis before surgery or imaging data for the purpose of characterizing the percentage of necrosis.

**Table 3:** Description of local therapies (*n* = 43).

Local Treatment	Patients, *n* (%)
Surgery	18 (41.9)
Complete	10 (23.3)
Incomplete	8 (18.6)
Radiotherapy	
SRS	8 (18.6)
WBRT	5 (11.6)
Post-surgery SRS	12 (27.9)
Timing between radiotherapy and ICI administration	
Radiotherapy before ICI	12 (48)
Concomitant	13 (52)
Delay between radiotherapy and start of ICI, weeks, Median (range)	1 (1–4)
Brain response after local therapy and before ICI	
CR	10 (40)
PR	9 (36)
SD	0 (0)
PD	3 (12)
Death before new imaging	3 (12)

Abbreviations: CR, complete response; ICI, immune checkpoint inhibitor; PD, progressive disease; PR, partial response; SD, stable disease; SRS, stereotaxic radiotherapy; WBRT, whole-brain radiotherapy.

**Table 4:** Efficacy on brain metastasis (BM).

Characteristics	Patients, *n* (%)
Delay between diagnosis and start of ICI, days, Median (range)	36 (3–132)
Best responses characteristics for BM according to RECIST criteria	
CR	15 (34.9)
PR	13 (30.2)
SD	3 (7)
PD	12 (27.9)
Best responses characteristics for BM according to RANO criteria	
CR	16 (37.2)
PR	10 (23.3)
SD	3 (7)
PD	14 (32.6)
Number of cycles before best responses ^†^, Median (range)	5 (1–13)
Shrinkage of BM after start of ICI, Median (range)	51 (0–100)
Shrinkage of brain edema after start of ICI, Median (range)	82 (−6–100)
Type of progressive disease, n (%) ^∆^	
New BM	2 (20)
Existing BM	4 (40)
Both	4 (40)
Progression free survival, month, Median [95% CI]	8.3 [3.0; 13.8]
Cerebral progression free survival, month, Median [95% CI]	5.3 [3.0; 14.7]
Systemic progression free survival, month, Median [95% CI]	9.2 [4.5; 17.3]
Dissociate response between BM and extracerebral metastasis	
No	3 (11.5)
Only systemic progression	16 (61.5)
Only cerebral progression	7 (26.9)
Reason of definitive interruption of ICI ^‖^	
Progressive disease	15 (55.6)
Adverse event	4 (14.8)
Death	3 (11.1)
Other	5 (18.5)
Overall survival, month, Median [95% CI]	25.5 [9.8–NR]

Abbreviations: BM, brain metastasis; CR, complete response; IC, immune checkpoint inhibitor; PD, progressive disease; PR, partial response; SD, stable disease. Notes: ^†^ N = 22 patients; 3 patients never progressed after surgery before ICI therapy; ^∆^ N = 10 patients whose cerebral progression was assessed; ^‖^ N = 27 patients whose treatment was interrupted before the end of the study.

**Table 5:** Multivariate analysis of prognostic factors for progression-free survival.

Characteristics	HR (95% CI)
Surgery	0.327 [0.138; 0.770] (*p* = 0.011)
Ratio of cumulative size of BM (mm)/number of BM	1.050 [1.008; 1.093] (*p* = 0.018)

## Data Availability

The datasets analyzed during the current study are available from the corresponding author upon reasonable request.

## Supplementary Materials

The following supporting information can be downloaded at: https://www.mdpi.com/article/10.3390/curroncol31030126/s1, Table S1: General characteristics of patients following treatment with pembrolizumab (IO) (*n* = 43); Table S2: Univariate analysis of prognostic factors for cerebral progression-free survival (cPFS); Table S3: Univariate analysis of prognostic factors for progression-free survival (PFS).
