# Supplementary material for: Outcomes of Patients with Non-Small Cell Lung Cancer and Brain Metastases Treated with the Upfront Single Agent Pembrolizumab: A Retrospective and Multicentric Study of the ESCKEYP GFPC Cohort
Source: Curr Oncol. 2024 Mar 21;31(3):1656–66. doi: 10.3390/curroncol31030126 (PMC10969391; doi:10.3390/curroncol31030126)
Supplement: Supplementary file 1 [file curroncol-31-00126-s001.zip › curroncol-2891992-supplementary.pdf]

Current Oncology MDPI

Supplementary Material

Table S1: General characteristics of patients following treatment with pembrolizumab (IO) (n = 43).

Characteristics	Population, n (%)
Treatment after ICI	
Yes	22 (51.2)
No	21 (48.8)
Radiotherapy‡	
No	11 (50)
Brain radiotherapy	4 (18.2)
Extracerebral radiotherapy	7 (31.8)
Systemic treatment	
Chemotherapy	16 (37.2)
Targeted therapy	2 (4.7)
Number of cycles of systemic treatment^Δ^, Median (range)	7 (1–37)
Best tumoral response†	
CR	0 (0)
PR	7 (38.9)
SD	7 (38.9)
PD	4 (22.2)
Progression free survival after start of new systemic lines, months, Median [95% CI]	10.8 [4.23; 25.44]

Abbreviations: CR, complete response; ICI, immune checkpoint inhibitor; PD, progressive disease; PR, partial response; SD, stable disease

Notes:

‡N = 22 patients who received treatment following ICI therapy

ΔN = 18 patients who received systemic treatment following ICI therapy

†N = 18 patients, one patient died before evaluation, and three patients had missing data about the evaluation of the new therapeutic line

Table S2: Univariate analysis of prognostic factors for cerebral progression-free survival (cPFS).

Characteristics	HR (95%CI)
Age	0.967 [0.901; 1.038]
Surgery (yes vs no)	1.118 [0.311; 4.015]
Radiotherapy (yes vs no)	2.666 [0.563; 12.620]
Number of metastases	0.981 [0.770; 1.249]
Ratio of cumulative size (mm)/number of BM	1.078 [1.022; 1.136] (p=0.006)
Necrosis (less or more than 50%)	2.033 [0.564; 7.325]
Smoking status (no smoker vs ancient or former smoker)	1.314 [0.161; 10.740]
Performance status	1.177 [0.299; 4.644]
Body mass index	1.060 [0.978; 1.150]
Side effect related to Pembrolizumab (yes vs no)	1.323 [0.340; 5.152]
Tumor PD-L1 expression	1.405 [0.396; 4.980]

Abbreviations: BM, brain metastasis ; vs, versus

Table S3: Univariate analysis of prognostic factors for progression-free survival (PFS).

Characteristics	HR (95%CI)
Age	0.997 [0.961; 1.034]
Surgery (yes vs no)	0.438 [0.226; 0.850] (p = 0.015)
Radiotherapy (yes vs no)	0.867 [0.460; 1.633]
Number of metastases	1.037 [0.935; 1.149]
Ratio of cumulative size (mm)/number of BM	1.032 [1.000; 1.066]
Necrosis (less or more than 50%)	1.440 [0.737; 2.813]
Smoking status (no smoker vs ancient or former smoker)	2.230 [0.846; 5.874]
Performance status	1.295 [0.957; 1.752]
Body mass index	1.031 [0.978; 1.088]
Side effect related to Pembrolizumab (yes vs no)	0.531 [0.278; 1.013]
Tumor PD-L1 expression	0.981 [0.519; 1.855]

Abbreviations: BM, brain metastasis ; vs, versus
